# Protective Effect of *Lactobacillus plantarum* P8 on Growth Performance, Intestinal Health, and Microbiota in *Eimeria*-Infected Broilers

**DOI:** 10.3389/fmicb.2021.705758

**Published:** 2021-07-09

**Authors:** Yang Wang, Xiaoguo Lv, Xuemin Li, Jinshan Zhao, Kai Zhang, Xiaojing Hao, Kaidong Liu, Huawei Liu

**Affiliations:** ^1^College of Animal Science and Technology, Qingdao Agricultural University, Qingdao, China; ^2^Qingdao Institute of Animal Science and Veterinary Medicine, Qingdao, China

**Keywords:** *Lactobacillus plantarum* P8, broiler, *Eimeria*-infected, intestinal immunity, gut microbiota

## Abstract

Coccidiosis is one of the major parasitic diseases in the commercial broiler industry. Probiotics can protect poultry against *Eimeria* infection. However, the mechanisms are not fully known. Therefore, *Lactobacillus plantarum* P8 (P8) was used to investigate its anti-coccidial property and mechanism. Five hundred broilers were allocated to five treatments: control diet (NC), control diet + *Eimeria* infection (IC), control diet containing 1 × 10^7^ cfu/g P8 + *Eimeria* infection (P8L), control diet containing 1 × 10^8^ cfu/g P8 + *Eimeria* infection (P8H), and control diet + *Eimeria* infection + Diclazuril (DIC). At day 14, all treatments except NC were inoculated with sporulated oocysts. Results indicated that *Eimeria* infection increased the mortality and oocysts shedding, and declined the growth performance as well as the intestinal barrier in *Eimeria*-treated broilers. On the contrary, dietary supplementation of low level P8, high level P8 and DIC decreased the mortality and oocysts shedding, but improved the growth performance and intestinal barrier. The impaired intestinal morphology in the IC group was also improved by P8H and DIC treatments. Besides, the elevated oxidative stress and pro-inflammation in *Eimeria*-infected broilers were reduced by P8L, P8H, and DIC treatments. Metagenomic analysis indicated P8 altered the structure of the gut microbiota, and the alteration was more obvious at day 21 than day 42. Notably, IC also increased the abundances of Eimeriidae, *Eimeria* and *Eimeria tenella* at day 21, while P8L and DIC decreased the abundances. Correlation analysis revealed that bacteria in *Eimeria*-treated broilers positively correlated with the intestinal permeability, oxidative stress and inflammation, while bacteria in broilers receiving P8L and DIC negatively correlated with the aforementioned pathological indices. Functional prediction demonstrated that the metagenomes of *Eimeria*-infected broilers were involved in several diseases. But the metagenomes of P8L-treated broilers were involved in energy metabolism and replication repair. In conclusion, dietary P8 supplementation inhibited oocyst shedding and improved the growth performance as well as the intestinal health of broilers infected with *Eimeria*, which was closely related to the regulation of gut microbiota. Moreover, the effects of P8 may be more effective in the early infection of coccidia.

## Introduction

Coccidiosis is one of the major parasitic diseases in the commercial broiler industry. Although much medical and managerial progress has been employed, the incidence of coccidiosis in the marketable poultry (broiler) can range from 5 to 70%, due to higher stocking densities and improper management practices (Du and Hua, 2004; Nnadi and George, 2010; Shamim et al., 2015). *Eimeria tenella* (*E. tenella*), *Eimeria necatrix*, *Eimeria maxima*, and *Eimeria acervulina* are the main *Eimeria* species that cause coccidiosis and inhabit in the intestine of chickens, leading to impaired intestinal function and growth performance (Dalloul et al., 2003; Min et al., 2013; Ritzi et al., 2014). Conventional coccidiosis control strategies include anticoccidial drugs and vaccination. However, existing vaccines consist of live virulent or attenuated *Eimeria* strains with limited scope of protection against an ever-evolving and widespread pathogen (Giannenas et al., 2012). Moreover, the use of anticoccidial drugs may lead to drug-resistant *Eimeria* strains and public concerns over residual drug of meat and eggs (Bun et al., 2011; Giannenas et al., 2014). Thus, exploring alternative methods is urgent.

Probiotics are live microorganisms which when administrates in adequate amounts confer a health benefit on the host (Araya et al., 2002). In the past decades, studies have demonstrated that probiotics effectively protect poultry against *Eimeria* infection (Lee et al., 2007; Ritzi et al., 2014; El-Sawah et al., 2020). Although the protective mechanisms of probiotics are still not fully known, it has been established that strain-specific probiotics can significantly enhance the mucosa-associated immune responses, increase the production of anti-*Eimeria* antibody and decrease the oocyst output (Travers et al., 2011). *Lactobacillus plantarum* P8 (P8) is a probiotic strain isolated from the natural fermented yogurt of the Inner Mongolian herder’s family. Beneficial effects of P8, including the decrease of stress in stressed adults (Lew et al., 2019), the improvement of lipid metabolism in rats (Bao et al., 2012) and the regulation of intestinal morphology in juvenile turbots (Wang et al., 2016) were reported. However, whether P8 can attenuate *Eimeria* infection in broilers is still unknown. Thus, this study aimed to evaluate the effects of P8 on the growth performance, intestinal antioxidant capacity, immune response and morphology of broilers with coccidiosis. Moreover, despite the significant damage that *Eimeria* causes to the chicken gastrointestinal tract, little is known about its influence on the enteric microbiome, or whether the resident microbiota play any role in modulating parasite-induced pathology. Therefore, the roles of *Eimeria* and P8 in regulating gut microbiota of broilers were focused in this study.

## Materials and Methods

### Materials

The probiotic P8 powder (1 × 10^11^ cfu/g) was purchased from Beijing SciTop Bio-tech Co., Ltd. (Beijing, China). The anticoccidial drug Diclazuril (5%) (DIC) was obtained from Xinxiang Huachu Trading Co., Ltd. (Xinxiang, China). Four *Eimeria* species (*E. tenella*, *E. necatrix*, *E. maxima*, and *E. acervulina*) used in the present experiment were isolated and provided by the Parasitology Laboratory, College of Veterinary Medicine, Qingdao Agricultural University. It was maintained by periodic passage through coccidia-free chickens, and those unsporulated oocysts obtained from feces of day 5 post infection were purified and processed by standard operation. The degree of sporulation and oocysts population was enumerated by microscopy (Wang et al., 2008).

### Birds and Diets

Five hundred one-day-old male Arbor Acres broilers with similar initial body weights were purchased from Henan Academy of Agricultural Sciences. The basal diet (containing no anticoccidial drug) was obtained from Henan Academy of Agricultural Sciences. The composition and nutrient levels of the basal diet is listed in Table 1. The appropriate quantity of P8 was pre-mixed with 1 kg of the basal diet and successively mixed into the remaining diet to obtain the prefixed inclusion level at a batch of feed (Liu et al., 2018).

**TABLE 1:** Composition and nutrient levels of basal diets (air-dry basis) %.

Items	Contents	
	
	1–21 days of age	22–42 days of age
**Ingredients**		
Corn	60	62.00
Soybean meal	34.30	30.50
Soybean oil	2.00	4.00
Limestone	1.45	1.40
CaHPO_4_	1.33	1.28
Methionine	0.25	0.15
NaCl	0.35	0.35
Premix^a^	0.20	0.20
Multi-vitamin^b^	0.02	0.02
Choline chloride	0.10	0.10
Total Nutrient levels^c^		
Metabolizable energy (MJ/kg)	12.54	12.96
Crude protein	20.65	18.98
Calcium	1.00	0.90
Available phosphorus	0.45	0.40
Lysine	1.09	0.99
Methionine	0.56	0.44

*^*a*^Provided per kilogram of diet: Fe (as ferrous sulfate) 80 mg; Cu (as copper sulfate) 10 mg; Zn (as zinc sulfate) 75 mg; Mn (as manganese sulfate) 80 mg; Se (as sodium selenite) 0.30 mg; I (as potassium iodide) 0.40 mg.*

*^*b*^Provided per kilogram of diet: Vitamin A (*trans*-retinyl acetate) 8000 IU; Vitamin D_3_ (cholecalciferol) 3000 IU; Vitamin E (all-rac-α-tocopherol acetate) 20 IU; Vitamin K_3_ (menadione) 2.0 mg; Vitamin B_1_ (thiamin) 4.2 mg; Vitamin B_2_ (riboflavin) 4.0 mg; Vitamin B_6_ (pyridoxine HCl) 4.5 mg; Vitamin B_12_ (cobalamin) 0.02 mg; nicotinic acid 10 mg; calcium pantothenate 11 mg; folic acid 1.0 mg; biotin 0.15 mg.*

*^*c*^The nutrient levels were calculated values.*

### Purity and Identification Checks of Bacteria

The culture and preparation of P8 was prepared by the Department of Animal Nutrition, Qingdao Agricultural University, China. P8 was cultured on Man Rogosa Sharpe media, kept at 37°C for 24 h. Pure bacterial cells were collected after centrifugation at 5000 × *g* for 10 min at 4°C. Then, these cells were washed twice with sterile 0.85% sodium chloride solution. Ultimately, the culture purity and identification were constantly checked by the spreading plate method (Nikoskelainen et al., 2003).

### Experimental Design

A total of 500 broilers were equally divided into 5 treatments with 10 replicated cages of 10 birds each for a 42-day feeding period. The treatments were control diet (non-infected control, NC), control diet + *Eimeria* infection (Infected control, IC), control diet containing 1 × 10^7^ cfu/g P8 + *Eimeria* infection (P8L), control diet containing 1 × 10^8^ cfu/g P8 + *Eimeria* infection (P8H), and control diet + *Eimeria* infection + 0.2 g/kg anticoccidial drug (DIC). At day 14, all treatments except NC were inoculated with 1 mL saline containing 4 × 10^4^ sporulated oocysts (*E. tenella* 1 × 10^4^, *E. necatrix* 1 × 10^4^, *E. maxima* 1 × 10^4^, and *E. acervulina* 1 × 10^4^) by oral gavage (Yi et al., 2005), while broilers in the NC group inoculated esophageally with 1 mL saline. Fresh water and feed were provided *ad libitum*. The temperature of the room was set at 33–35°C in the first week, and then decreased 2°C every week until 24°C. The animal experiment was approved and performed in accordance with the guidelines of Ethics and Animal Welfare Committee of Qingdao Agricultural University.

### Measurement of Fecal Oocyst Enumeration, Cecal Lesion and Bloody Diarrhea

Excreta samples of birds from each replicate were observed and collected on day 19 (5 days after challenge infection), and the average of bloody diarrheal score (BDS) and the number of fecal oocysts was determined according to the method described by Youn and Noh (2001) and Cha et al. (2018), respectively. The cecal lesion score (CLS) from 6 cecal samples per group were evaluated according to Johnson and Reid (1970).

### Measurement of Growth Performance

The amounts of provided and refused feed were measured daily on a replicate basis to calculate the average daily feed intake (ADFI). Body weight (BW) was measured at days 0, 14, and 42 to calculate average daily gain (ADG) and the feed:gain ratio (F:G) on a replicate basis. Mortality was recorded daily.

### Sample Collection

At days 21 and 42, blood samples from 1 broiler of each replicate were randomly collected by cardiac puncture into vacuum tubes containing coagulant and centrifuged for 10 min (3000 × *g*) at 4°C. Pure serum samples were collected and stored in 1.5 mL Eppendorf tubes at −20°C. The segments of duodenum, jejunum and ileum from 1 broiler of each replicate were collected and fixed in 10% buffered formaldehyde for 24 h. Mucosa was scraped from 10 cm of the jejunum using a glass slide (5 cm proximal to the Meckel’s diverticulum) from 1 broiler of each replicate. Six cecal samples from each group were collected for cecal lesion analysis. At day 21, the cecal contents of 6 broilers per treatment were collected for Illumina Sequencing. At day 42, the cecal contents of 8 broilers per treatment were collected for Illumina Sequencing. Cecal contents and all intestinal samples except the intestine segments in buffered formaldehyde were placed immediately in liquid nitrogen and then held at −80°C.

### Analysis of Biochemical Indices

The level of malonaldehyde (MDA) and the activities of superoxide dismutase (SOD), catalase (CAT), glutathione peroxidase (GPX) and total antioxidant capacity (T-AOC) in the jejunal mucosa were determined spectrophotometrically using commercial kits (Suzhou Grace Biotechnology Co., Ltd.) according to manufacturer’s protocol. The levels of sIgA, interleukin 6 (IL-6), IL-10 and tumor necrosis factor (TNF-α) in the jejunal mucosa, and the levels of diamine oxidase and D-lactate in the serum were determined using ELISA kits (Shanghai Enzyme-linked Biotechnology Co., Ltd.) according to manufacturer’s protocol.

### Intestinal Morphology

The intestinal segments were embedded in paraffin, and the section of each sample was placed on a glass slide and stained with hematoxylin–eosin (HE). The villus was observed under an OLYMPUS microscope (OLYMPUS, Japan) using the HMIAS-2000 image analysis system. Villus height was measured from the top of the villus to the villus crypt junction, and crypt depth was measured as the depth of the invagination between adjacent villus (Shan et al., 2019).

### Analysis of Tight Junction Proteins

The protein of six jejunal mucosa samples from each group was extracted by Nuclear and Cytoplasmic Protein Extraction Kit (Beyotime Biotechnology, China) according to manufacturer’s protocol. Equal amounts of proteins from each sample were subjected to SDS-PAGE, then proteins on the gel were transferred to nitrocellulose membrane. Membranes were blocked by 5% skimmed milk and then incubated with the primary antibodies (anti-β-actin, anti-Claudin-1, and anti-Occludin) overnight at 4°C. After washing with Tris Buffered Saline Tween, membranes were incubated with secondary antibody adjusted with Horseradish Peroxidase (Beyotime Biotechnology, China) (Wang et al., 2017). The blots were then developed with an electrochemiluminescence detection system according to the manufacturer’s instruction. Densitometric quantification of band intensities was determined using ImageJ software (National Institutes of Health, United States).

### DNA Extraction and Metagenomic Analysis

Bacterial DNA from 6 cecal contents (day 21) and 8 cecal contents (day 42) was extracted using a TIANamp stool DNA kit (Tiangen Biotech Co. Ltd., Beijing) according to the manufacturer’s instruction. DNA samples were quantified using a Qubit 2.0 Fluorometer (Invitrogen, United States) and DNA quality was confirmed using 0.8% agarose gel electrophoresis. Purified DNA was sent to Novogene Biotech Co., Ltd. (Beijing, China) for Illumina MiSeq sequencing. A library consisting 300 bp paired-end reads were generated before sequencing. After removing sequences contaminated by N bases, adapter sequences, low quality sequences, and replicate sequences, according to the quality control pipeline recommended by Beijing Genome Institute (BGI, Shenzhen, China), the quality-filtered reads were obtained for subsequent metagenomic analysis of cecal contents from broilers (Tong et al., 2017).

Quality filtering of the raw tags was performed to generate high-quality clean tags according to QIIME (Quantitative Insights Into Microbial Ecology, version 1.2.1^1^). Operational taxonomic units (OTUs) were clustered at 97% sequence similarity following the Uclust (version 1.2.22^2^), and representative sequences of each cluster were used to assign taxonomy through annotation against the SILVA database. The alpha diversity of the samples, Chao 1, Ace, Shannon, Invisimpson, and Simpson indices were evaluated. Principal coordinate analysis (PCoA) of the OTUs in different groups was conducted using R version 3.5.1 Feather Spray^3^. The prediction of the functional genes in the gut microbiota was done using the protocol from PICRUSt. A closed-reference OTU table in a biom-format from the script *pick_closed_reference_otus.py* generated in QIIME was used. The taxonomy assignment was made with the reference sequences from Greengenes database v13.8 with a 97% similarity. After that, the OTU table was normalized with the PICRUSt workflow using the Langille Lab Online Galaxy Instance^4^ to obtain the final metagenome functional prediction from the Kyoto Encyclopedia of Genes and Genomes (KEGG) database at hierarchy level 1 pathways. Finally, the Statistical Analysis of Metagenomic Profiles (STAMP) software v2.1.3 was used to analyze the PICRUSt-predicted metagenomes in order to obtain significant differences in the functional genes between the groups using the Kruskal–Wallis test, and we used Storey’s FDR approach for multiple test correction (Chavez-Carbajal et al., 2019). All the DNA datasets have been submitted to the NCBI Sequence Read Archive database under the BioProject ID: PRJNA683158.

### Statistical Data Analysis

One-way ANOVA was used for single factor analysis by SPSS 20.0 for windows (SPSS Inc., Chicago, IL, United States). Spearman’s correlation coefficient was calculated using SPSS Version 20.0 (SPSS Inc., Chicago, IL, United States) and GraphPad Prism 8 (GraphPad Software, Inc.) software and used to assess bivariate relationships between variables. Results were expressed as means and the differences were considered significant at *P* < 0.05.

## Results

### Effects of P8 on the Fecal Oocyst Shedding, Bloody Diarrhea and Cecal Lesion of *Eimeria*-Infected Broilers

At the 5th day post infection, *Eimeria* significantly increased the oocysts per gram of excreta (OPG) (*P* < 0.01). Whereas, dietary supplementation with low level P8, high level P8 and DIC significantly decreased the OPG compared with the IC group (*P* < 0.01). Notably, P8H and DIC treatments resulted in lower OPG than the P8L treatment (*P* < 0.01). Furthermore, the BDS and CLS of broilers were significantly elevated in the IC group compared with the NC group (*P* < 0.01). In comparison to the IC group, the P8L, P8H and DIC treatments significantly lowered the BDS and CLS (*P* < 0.01). Besides, infected broilers receiving DIC had decreased BDS and CLS compared to that receiving different doses of P8 (*P* < 0.01) (Table 2).

**TABLE 2:** Effects of P8 on the fecal oocyst shedding, bloody diarrhea and cecal lesion of *Eimeria*-infected broilers.

	NC	IC	P8L	P8H	DIC	SEM	*P*-value
**5 days after infection**							
OPG (×10^5^/g of excreta)	0.00^c^	11.96^a^	4.52^b^	1.28^c^	0.55^c^	0.723	0.001
BDS	0.00^c^	1.72^a^	1.18^b^	0.91^b^	0.18^c^	0.108	0.001
CLS	0.00^c^	2.36^a^	1.09^b^	1.27^b^	0.40^c^	0.137	0.001

*^*a,b,c*^Mean value within a role with no common superscript differ significantly (*P* < 0.05). NC, control diet; IC, control diet + *Eimeria* infection; P8L, control diet containing 1 × 10^7^ cfu/g P8 + *Eimeria* infection; P8H, control diet containing 1 × 10^8^ cfu/g P8 + *Eimeria* infection; DIC, control diet + *Eimeria* infection + Diclazuril. OPG, oocysts per gram of excreta. BDS, bloody diarrheal score. CLS, cecal lesion score.*

### Effects of P8 on the Growth Performance of *Eimeria*-Infected Broilers

No significant changes were observed for the growth performance among groups during days 0 to 14. However, *Eimeria*-infection significantly decreased the ADG (*P* < 0.01) and increased the F:G (*P* < 0.05) compared to the NC group during days 14 to 42. On the contrary, in comparison to the IC group, P8L, P8H, and DIC treatments significantly increased the ADG (*P* < 0.01) and decreased the F:G (*P* < 0.05) during days 14 to 42. In addition, compared to the NC group, the IC treatment increased the mortality significantly (*P* < 0.05), however, P8L, P8H, and DIC treatments decreased the mortality significantly compared to the IC group (*P* < 0.05) (Table 3).

**TABLE 3:** Effects of P8 on the growth performance of *Eimeria*-infected broilers.

	NC	IC	P8L	P8H	DIC	SEM	*P*-value
**Days 0–14**							
ADG (g)	23.07	22.98	24.05	24.01	24.06	0.256	0.405
ADFI (g)	33.72	34.57	35.87	35.81	35.75	0.349	0.216
F:G	1.47	1.51	1.49	1.49	1.48	0.020	0.917
**Days 14–42**							
ADG (g)	58.21^a^	47.48^c^	51.85^b^	52.01^b^	52.37^b^	0.721	0.000
ADFI (g)	108.62	100.25	100.73	102.46	104.65	1.345	0.431
F:G	1.87^c^	2.11^a^	1.94^b^	1.97^b^	2.00^b^	0.021	0.012
Mortality	2.00^b^	11.00^a^	2.00^b^	1.00^b^	0.00^b^	1.398	0.022

*^*a,b,c*^Mean value within a role with no common superscript differ significantly (*P* < 0.05). NC, control diet; IC, control diet + *Eimeria* infection; P8L, control diet containing 1 × 10^7^ cfu/g P8 + *Eimeria* infection; P8H, control diet containing 1 × 10^8^ cfu/g P8 + *Eimeria* infection; DIC, control diet + *Eimeria* infection + Diclazuril. ADG, average daily gain. ADFI, average daily feed intake. F:G, feed:gain ratio.*

### Effects of P8 on the Intestinal Morphology of *Eimeria*-Infected Broilers

**FIGURE 1:**
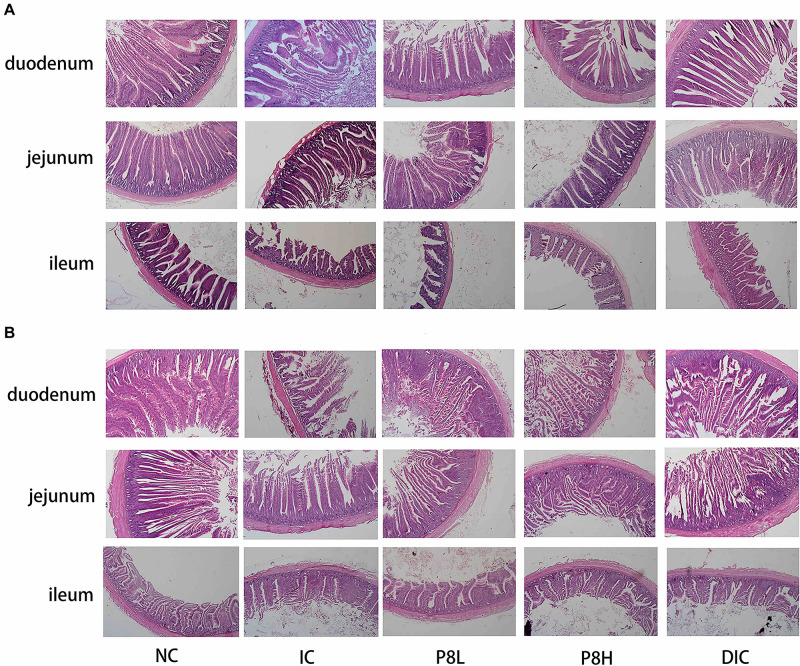
Effects of P8 on the intestinal morphology of *Eimeria*-infected broilers. **(A)** Day 21, **(B)** day 42.

At day 21, duodenal HE staining sections showed that the IC treatment significantly decreased the villus height compared to the NC group (*P* < 0.01), however, P8H and DIC treatments significantly increased the villus height compared to the IC treatment (*P* < 0.01). Although the crypt depth was not obviously changed among groups, the IC treatment significantly decreased the villus height/crypt depth (V/C) value (*P* < 0.05), which was significantly reversed by the DIC treatment (*P* < 0.05). As for the jejunum, the IC treatment led to the lower villus height (*P* < 0.01) and V/C value (*P* < 0.05) compared to the NC group, however, P8H and DIC treatments increased the villus height (*P* < 0.01) and V/C value (*P* < 0.05) compared to the IC treatment. There were no significant impacts of the different treatments on the tested morphology parameters in the ileum (Figure 1A and Table 4). At day 42, compared to the NC group, the IC treatment significantly decreased the villus height and V/C value (*P* < 0.01) of duodenum, but compared to the IC group, broilers in P8H and DIC groups had the increased villus height (*P* < 0.01). Moreover, the jejunal and ileal villus heights were also decreased in the IC group compared to the NC group (*P* < 0.01). Nevertheless, in comparison to the IC treatment, the DIC treatment effectively increased the villus height (*P* < 0.01). Besides, compared to the NC group, the jejunal V/C value was also down-regulated in broilers with coccidiosis (*P* < 0.05), but the DIC treatment elevated V/C value significantly compared to that of the IC treatment (*P* < 0.05) (Figure 1B and Table 4).

**TABLE 4:** Effects of P8 on the intestinal morphology of *Eimeria*-infected broilers at days 21 and 42.

	NC	IC	P8L	P8H	DIC	SEM	*P*-value
**Day 21**							
**Duodenum**							
Villus height (μm)	783.62^a^	628.23^d^	653.17^cd^	691.53^bc^	723.37^b^	15.38	0.001
Crypt depth (μm)	113.02	104.62	104.62	101.69	106.24	1.97	0.410
V/C	7.23^a^	6.01^b^	6.44^ab^	6.53^ab^	7.11^a^	0.14	0.028
**Jejunum**							
Villus height (μm)	316.64^a^	263.57^c^	285.14^bc^	301.84^ab^	309.42^ab^	7.65	0.000
Crypt depth (μm)	65.56	75.96	70.58	69.98	68.46	3.71	0.147
V/C	4.83^a^	3.47^c^	4.04^bc^	4.31^ab^	4.52^ab^	0.19	0.028
**Ileum**							
Villus height (μm)	185.93	139.42	165.18	174.48	191.95	6.81	0.087
Crypt depth (μm)	50.13	48.62	51.78	52.07	50.95	1.84	0.985
V/C	3.75	2.95	3.25	3.41	3.82	0.17	0.546
**Day 42**							
**Duodenum**							
Villus height (μm)	940.07^a^	765.18^d^	790.52^d^	818.21^c^	856.36^b^	16.61	0.001
Crypt depth (μm)	151.07	154.20	160.87	158.93	161.93	2.20	0.530
V/C	6.22^a^	4.96^b^	4.92^b^	5.16^b^	5.32^b^	0.14	0.003
**Jejunum**							
Villus height (μm)	617.64^a^	554.80^cd^	527.95^d^	571.95^bc^	598.18^ab^	9.32	0.001
Crypt depth (μm)	102.13	106.16	102.02	100.67	100.52	0.94	0.350
V/C	6.05^a^	5.23^b^	5.17^b^	5.68^ab^	5.95^a^	0.11	0.011
**Ileum**							
Villus height (μm)	391.71^a^	336.55^b^	349.42^b^	355.03^b^	386.08^a^	6.14	0.001
Crypt depth (μm)	53.96	49.77	54.49	51.78	54.77	2.09	0.955
V/C	7.29	6.91	6.84	6.94	7.11	0.29	0.993

*^*a,b,c,d*^Mean value within a role with no common superscript differ significantly (*P* < 0.05). NC, control diet; IC, control diet + *Eimeria* infection; P8L, control diet containing 1 × 10^7^ cfu/g P8 + *Eimeria* infection; P8H, control diet containing 1 × 10^8^ cfu/g P8 + *Eimeria* infection; DIC, control diet + *Eimeria* infection + Diclazuril. V/C, ratio between villus height and crypt depth.*

### Effects of P8 on the Expressions of Jejunal Tight Junction Proteins and the Levels of D-Lactate and Diamine Oxidase of *Eimeria*-Infected Broilers

**FIGURE 2:**
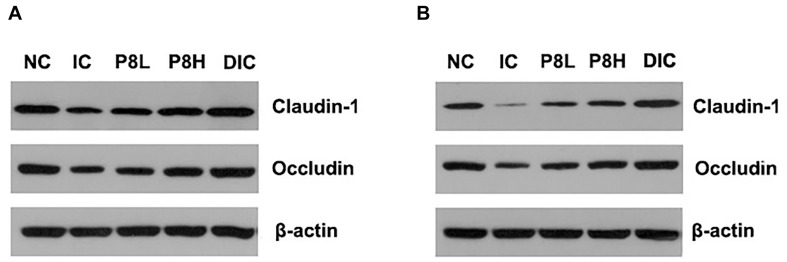
Effects of P8 on the expression of jejunal Claudin-1 and Occludin of *Eimeria*-infected broilers. **(A)** Day 21, **(B)** day 42.

The expressions of tight junction proteins Claudin-1 and Occludin are shown in Figure 2 and Table 5. At day 21, Claudin-1 and Occludin expressions in the IC group were significantly decreased compared to the NC group (*P* < 0.01). However, P8L and P8H treatments significantly increased the expressions of Claudin-1 and Occludin compared to the IC group (*P* < 0.01). Besides, the DIC treatment induced the highest Claudin-1 and Occludin expressions among groups (*P* < 0.01). At day 42, the expressions of Claudin-1 and Occludin were also significantly reduced in broilers with the IC treatment compared to that of the NC treatment (*P* < 0.01), however, P8L, P8H, and DIC treatments significantly up-regulated the Claudin-1 and Occludin expressions compared to the IC treatment (*P* < 0.01). Furthermore, the serum D-lactate and diamine oxidase levels were significantly elevated in the IC group at days 21 and 42 (*P* < 0.01) compared to the NC group, however, P8L, P8H, and DIC treatments effectively declined the levels of D-lactate at days 21 and 42 (*P* < 0.01) compared to the IC treatment. The diamine oxidase levels were also reduced by P8L, P8H, and DIC treatments at day 21 (*P* < 0.01), but only reduced by P8H and DIC treatments at day 42 (*P* < 0.01) (Table 5).

**TABLE 5:** Effects of P8 on the expressions of intestinal tight junction proteins and levels of D-lactate and diamine oxidase of *Eimeria*-infected broilers at days 21 and 42.

	NC	IC	P8L	P8H	DIC	SEM	*P*-value
**Day 21**							
Claudin-1	0.788^b^	0.366^c^	0.673^b^	0.787^b^	0.970^a^	0.042	0.000
Occludin	0.755^b^	0.380^d^	0.560^c^	0.761^b^	0.923^a^	0.038	0.000
D-lactate (μmol/L)	53.76^b^	77.82^a^	62.67^b^	56.24^b^	61.91^b^	2.031	0.001
Diamine oxidase (ng/ml)	19.55^c^	30.68^a^	24.64^b^	22.83^b^	21.88^bc^	0.706	0.000
**Day 42**							
Claudin-1	0.474^ab^	0.201^c^	0.368^b^	0.502^ab^	0.594^a^	0.032	0.000
Occludin	0.629^ab^	0.282^d^	0.452^c^	0.548^bc^	0.725^a^	0.035	0.000
D-lactate (μmol/L)	49.87^b^	76.88^a^	56.78^b^	53.89^b^	53.01^b^	2.268	0.000
Diamine oxidase (ng/ml)	16.89^b^	24.50^a^	20.77^ab^	17.22^b^	17.78^b^	0.712	0.001

*^*a,b,c,d*^Mean value within a role with no common superscript differ significantly (*P* < 0.05). NC, control diet; IC, control diet + *Eimeria* infection; P8L, control diet containing 1 × 10^7^ cfu/g P8 + *Eimeria* infection; P8H, control diet containing 1 × 10^8^ cfu/g P8 + *Eimeria* infection; DIC, control diet + *Eimeria* infection + Diclazuril.*

### Effects of P8 on the Jejunal Antioxidant Capacity of *Eimeria*-Infected Broilers

At day 21, different treatments had no significant effects on CAT activity. Compared to the NC group, the IC treatment decreased the activities of T-AOC (*P* < 0.05), GPX (*P* < 0.05), and SOD (*P* < 0.01). However, compared to the IC group, the P8L treatment did not alter the activities of aforementioned antioxidases, but broilers in P8H and DIC groups had increased SOD activities (*P* < 0.01). In addition, compared to the NC group, the IC treatment induced a higher MDA level (*P* < 0.01), but P8L, P8H, and DIC treatments reduced the MDA level significantly (*P* < 0.01). At day 42, different treatments had no significant effects on CAT and GPX activities. But compared to the NC group, the IC treatment decreased T-AOC and SOD activities (*P* < 0.05). Compared to the IC group, the DIC supplementation significantly increased T-AOC and SOD activities (*P* < 0.05), nevertheless, P8 treatments had no significant effects on SOD activity, while the P8H treatment could increase the activity of T-AOC (*P* < 0.05). Additionally, the *Eimeria* infection increased the MDA level (*P* < 0.01), but P8L, P8H and DIC treatments had reversion effects of this change (*P* < 0.01) (Table 6).

**TABLE 6:** Effects of P8 on the antioxidant capacity in jejunum mucosa of *Eimeria*-infected broilers at days 21 and 42.

	NC	IC	P8L	P8H	DIC	SEM	*P*-value
**Day 21**							
T-AOC (U/mg protein)	0.99^a^	0.80^b^	0.81^b^	0.88^ab^	0.92^ab^	0.028	0.042
CAT (U/mg protein)	16.26	14.37	14.29	14.94	15.64	0.303	0.182
GPX (U/mg protein)	4.91^a^	3.91^b^	4.05^b^	4.34^ab^	4.36^ab^	0.101	0.015
SOD (U/mg protein)	10.03^a^	6.93^c^	8.18^bc^	9.13^ab^	9.24^ab^	0.296	0.008
MDA (nmol/mg protein)	14.77^b^	27.21^a^	16.41^b^	14.26^b^	15.37^b^	0.874	0.000
**Day 42**							
T-AOC (U/mg protein)	0.91^a^	0.61^c^	0.72^bc^	0.82^ab^	0.79^ab^	0.031	0.010
CAT (U/mg protein)	15.45	14.54	14.25	14.69	15.76	0.282	0.399
GPX (U/mg protein)	4.60	4.48	4.11	4.33	4.52	0.174	0.922
SOD (U/mg protein)	10.44^a^	7.91^b^	8.93^ab^	9.48^ab^	10.42^a^	0.341	0.049
MDA (nmol/mg protein)	11.78^b^	15.85^a^	11.02^b^	10.20^b^	10.57^b^	0.531	0.002

*^*a,b,c*^Mean value within a role with no common superscript differ significantly (*P* < 0.05). NC, control diet; IC, control diet + *Eimeria* infection; P8L, control diet containing 1 × 10^7^ cfu/g P8 + *Eimeria* infection; P8H, control diet containing 1 × 10^8^ cfu/g P8 + *Eimeria* infection; DIC, control diet + *Eimeria* infection + Diclazuril. T-AOC, total antioxidant capacity. CAT, catalase. GPX, glutathione peroxidase. SOD, superoxide dismutase. MDA, malondialdehyde.*

### Effects of P8 on the Jejunal Immunity of *Eimeria*-Infected Broilers

At day 21, compared to the NC group, the IC treatment significantly increased the IL-6 level (*P* < 0.01), but P8L, P8H, and DIC treatments significantly decreased the IL-6 level (*P* < 0.01). Moreover, *Eimeria* infection significantly decreased the IL-10 level but increased the TNF-α secretion compared to the NC group (*P* < 0.01). However, broilers receiving P8L, P8H, and DIC showed lowered TNF-α levels compared to that of the IC group (*P* < 0.01). *Eimeria* infection also induced a higher sIgA level compared to the NC group (*P* < 0.05). But neither P8 nor DIC treatment reversed the sIgA secretion to normal. At day 42, all treatments had no obvious effects on the level of IL-10. Nevertheless, the IC treatment increased the IL-6 level compared to the NC group (*P* < 0.01), but P8 or DIC supplementation did not alter the IL-6 level significantly compared to the IC group. Besides, in comparison to the NC group, *Eimeria* infection also induced a higher TNF-α level (*P* < 0.01), which was significantly decreased in P8L, P8H, and DIC groups (*P* < 0.01). Moreover, similar to day 21, the up-regulated sIgA level in the IC group (*P* < 0.05) was not significantly altered by the treatments of high level P8 and DIC (Table 7).

**TABLE 7:** Effects of P8 on the immune indices in jejunum mucosa of *Eimeria*-infected broilers at days 21 and 42.

	NC	IC	P8L	P8H	DIC	SEM	*P*-value
**Day 21**							
IL-6 (pg/mg protein)	5.50^b^	7.23^a^	6.27^b^	5.40^b^	5.57^b^	0.172	0.001
IL-10 (pg/mg protein)	29.94^a^	18.53^b^	18.47^b^	18.89^b^	20.34^b^	0.695	0.000
TNF-α (pg/mg protein)	2.46^c^	3.85^a^	2.91^b^	2.33^c^	3.17^b^	0.095	0.000
sIgA (ng/mg protein)	692.66^b^	852.38^a^	761.31^ab^	851.54^a^	778.99^ab^	20.849	0.044
**Day 42**							
IL-6 (pg/mg protein)	5.13^bc^	6.76^a^	6.00^ab^	5.43^bc^	4.98^b^	0.157	0.001
IL-10 (pg/mg protein)	22.06	19.27	19.74	20.31	20.35	0.580	0.634
TNF-α (pg/mg protein)	2.06^c^	3.41^a^	2.82^b^	2.40^bc^	2.15^c^	0.098	0.000
sIgA (ng/mg protein)	741.19^b^	860.75^a^	808.05^ab^	879.46^a^	851.95^a^	16.444	0.032

*^*a,b,c*^Mean value within a role with no common superscript differ significantly (*P* < 0.05). NC, control diet; IC, control diet + *Eimeria* infection; P8L, control diet containing 1 × 10^7^ cfu/g P8 + *Eimeria* infection; P8H, control diet containing 1 × 10^8^ cfu/g P8 + *Eimeria* infection; DIC, control diet + *Eimeria* infection + Diclazuril. IL-6, interleukin 6. IL-10, interleukin 10. TNF-α, tumor necrosis factor. sIgA, secretory immunoglobulin A.*

### Effects of P8 on the α-Diversity and β-Diversity of Gut Microbiota of *Eimeria*-Infected Broilers

**FIGURE 3:**
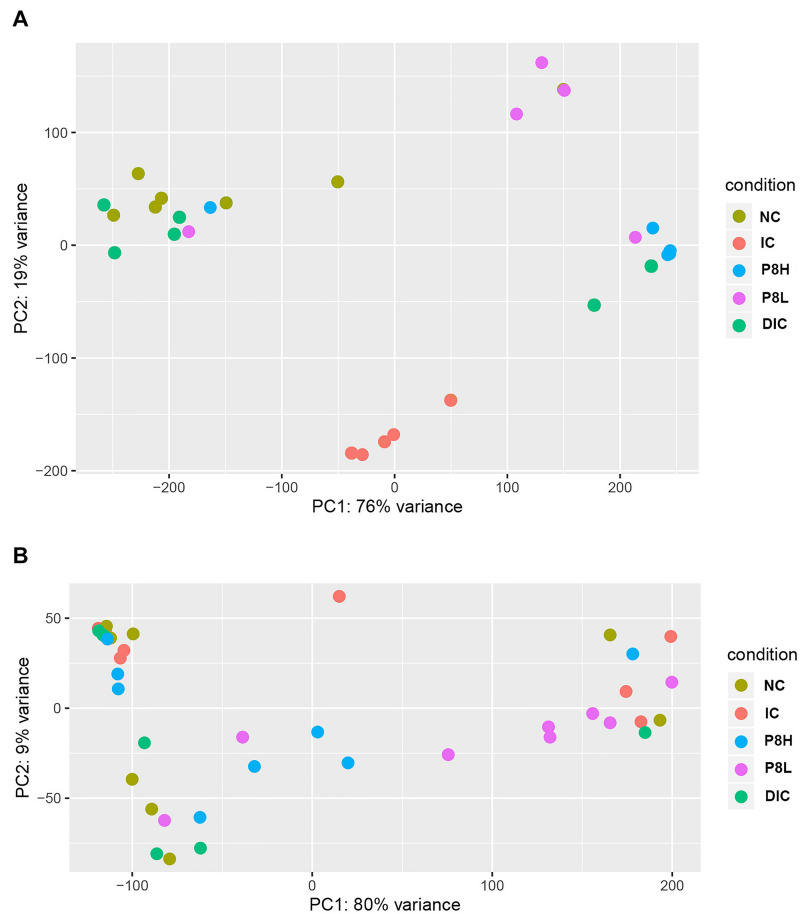
Effects of P8 on the PCoA of gut microbiota of *Eimeria*-infected broilers. **(A)** Day 21, **(B)** day 42.

At day 21, the IC treatment significantly declined the α-diversity parameters, such as Chao1 (*P* < 0.01), Ace (*P* < 0.01), Shannon (*P* < 0.05), and Invsimpson (*P* < 0.05) compared to the NC group. In addition, P8L and P8H treatments had no significant impacts on Chao1, Ace, Shannon, and Invsimpson, but the DIC treatment markedly elevated Chao1 and Ace (*P* < 0.01) compared to the IC treatment. All the different treatments had no significant effect on the Simpson parameter. Thereafter, at day 42, Chao1, Ace, and Shannon indices were not significantly altered by different treatments. In comparison to the IC treatment, P8L, P8H, or DIC treatment played no significant roles in regulating the values of Invsimpson and Simpson (Table 8). Moreover, as for the β-diversity, at day 21, the UniFrac PCoA revealed a distinct clustering of the gut microbiota composition between NC and IC groups (*P* < 0.05). Moreover, P8L, P8H, and DIC treatments also induced a distinct clustering of the gut microbiota compared to the IC treatment (*P* < 0.05) (Figure 3A). However, at day 42, no significant difference was observed for the clustering of the gut microbiota among groups (Figure 3B).

**TABLE 8:** Effects of P8 on the α-diversity of gut microbiota of *Eimeria*-infected broilers at days 21 and 42.

	NC	IC	P8L	P8H	DIC	SEM	*P*-value
**Day 21**							
Chao1	314.17^a^	85.83^c^	170.00^bc^	98.33^c^	269.17^ab^	25.52	0.005
Ace	314.17^a^	85.83^c^	170.00^bc^	98.33^c^	269.17^ab^	25.52	0.005
Shannon	2.23^a^	1.13^b^	1.44^ab^	1.12^b^	1.91^ab^	0.14	0.038
Invsimpson	3.81^a^	2.00^b^	2.19^b^	1.90^b^	3.06^ab^	0.22	0.014
Simpson	0.68	0.41	0.46	0.40	0.61	0.04	0.085
**Day 42**							
Chao1	424.50	404.50	437.63	384.63	436.00	9.32	0.327
Ace	424.50	404.50	437.63	384.63	436.00	9.32	0.327
Shannon	2.70	2.60	2.73	2.49	2.61	0.03	0.136
Invsimpson	4.70^a^	4.36^ab^	4.73^a^	4.04^b^	4.26^ab^	0.08	0.016
Simpson	0.79^a^	0.77^ab^	0.79^a^	0.75^b^	0.76^ab^	0.01	0.028

*^*a,b,c*^Mean value within a role with no common superscript differ significantly (*P* < 0.05). NC, control diet; IC, control diet + *Eimeria* infection; P8L, control diet containing 1 × 10^7^ cfu/g P8 + *Eimeria* infection; P8H, control diet containing 1 × 10^8^ cfu/g P8 + *Eimeria* infection; DIC, control diet + *Eimeria* infection + Diclazuril.*

### Effects of P8 on the Abundance of Gut Microbiota and Coccidia of *Eimeria*-Infected Broilers

**FIGURE 4:**
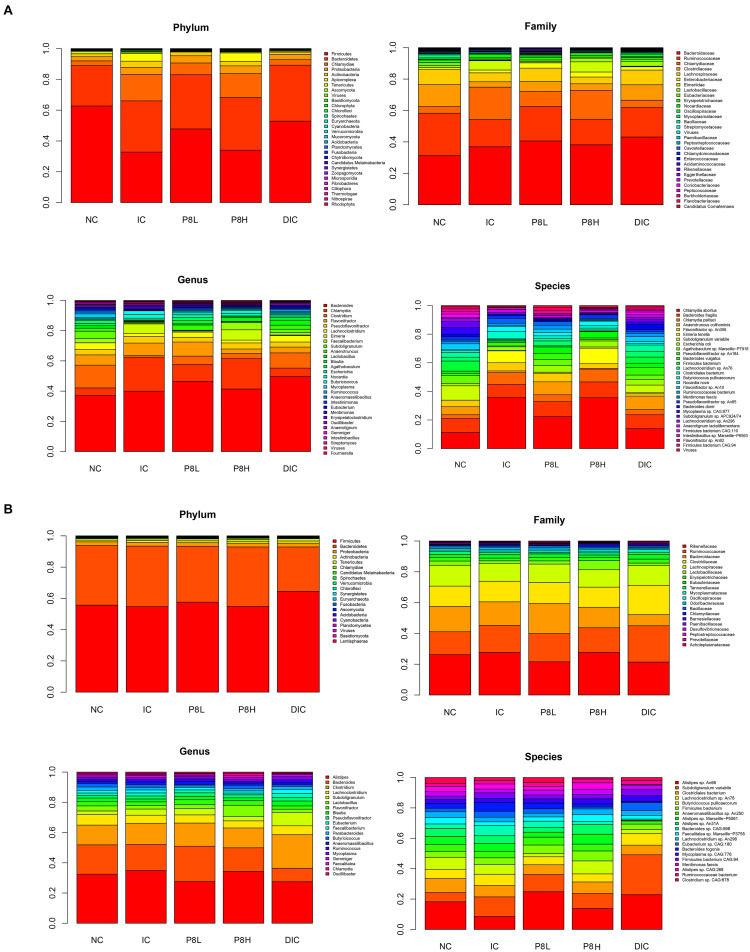
Effects of P8 on the abundance of gut microbiota and coccidia in broilers of *Eimeria*-infected broilers. **(A)** Day 21, **(B)** day 42.

The composition of the bacteria at phylum, family, genus and species levels were analyzed. At day 21, at phylum level, the abundances of Bacteroidetes and Proteobacteria and Actinobacteria were not altered significantly among groups. Nevertheless, compared to the NC group, broilers in the IC group had a significantly down-regulated Firmicutes abundance (*P* < 0.05) and a significantly up-regulated Chlamydiae abundance (*P* < 0.05). Then, compared to the IC group, the DIC treatment significantly decreased the Chlamydiae abundance (*P* < 0.05). Although P8L and DIC had no significant effects on Firmicutes and Chlamydiae abundances in comparison to the IC group, P8L and DIC could increase the Firmicutes abundance and decrease the Chlamydiae abundance to the levels that were not significantly different from the NC group. At family level, the abundances of the top 5 abundant bacteria (Bacteroidaceae, Ruminococcaceae, Chlamydiaceae, Clostridiaceae, and Lachnospiraceae) were not significantly changed by different treatments. At genus level, there were no significant differences in the abundances of *Bacteroides*, *Flavonifractor*, and *Pseudoflavonifractor* among groups. Additionally, the IC treatment led to elevated *Chlamydia* abundance (*P* < 0.05) and decreased *Clostridium* abundance (*P* < 0.01) compared to the NC group. Nevertheless, the DIC treatment significantly decreased *Chlamydia* (*P* < 0.05) and increased *Clostridium* (*P* < 0.01) compared to the IC treatment. At species level, IC, P8 or DIC treatment had no significant impacts on the abundances of *Bacteroides fragilis*, *Anaerotruncus colihominis*, and *Flavonifractor* sp. *An306.* However, the abundances of *Chlamydia abortus* and *Chlamydia psittaci* were increased by the IC treatment (*P* < 0.05), and the DIC treatment had reversion effects of the changes induced by the IC treatment (*P* < 0.05). Moreover, we also observed alterations for coccidia abundance. Specifically, IC treatment elevated Eimeriidae, *Eimeria* and *E. tenella* abundances (*P* < 0.01), which were significantly decreased in P8L and DIC groups (*P* < 0.01) (Figure 4A and Supplementary Table 1).

At day 42, no significant differences were noticed for the abundance of the major bacteria among groups at phylum, family, genus and species levels (Figure 4B and Supplementary Table 2).

### Effects of P8 on the Correlation Between Gut Microbiota, Coccidia and Biochemical Parameters

**FIGURE 5:**
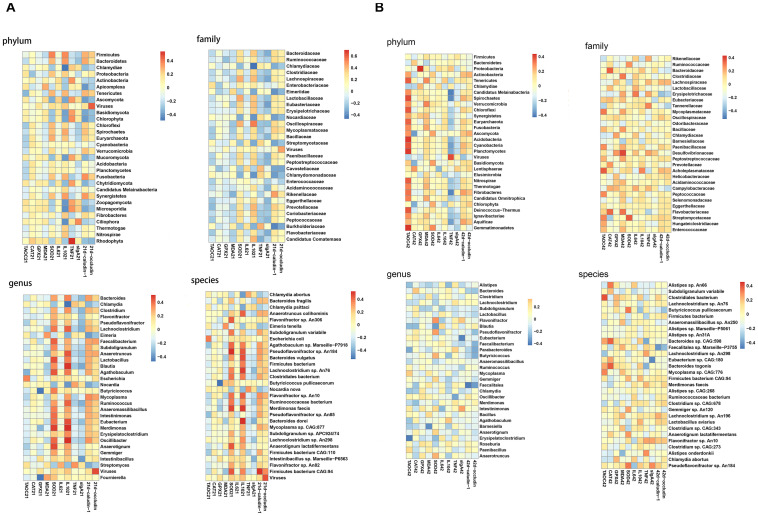
Predicted function of metagenomic genes in the cecal contents of *Eimeria*-infected broilers. **(A)** Day 21, **(B)** day 42.

The correlation between gut microbiota, coccidia and biochemical parameters was demonstrated in Figure 5. At day 21, at the phylum level, the jejunal SOD activity positively correlated with Firmicutes (*r* = 0.373, *P* < 0.01). The jejunal IL-10 level also positively correlated with Firmicutes (*r* = 0.366, *P* < 0.01), but negatively correlated with Chlamydiae (*r* = −0.468, *P* < 0.01). Moreover, jejunal TNF-α level positively correlated with Actinobacteria (*r* = 0.352, *P* < 0.01). At family level, jejunal SOD activity positively correlated with Lachnospiraceae (*r* = 0.424, *P* < 0.01). Besides, jejunal MDA level positively correlated with Eimeriidae (*r* = 0.424, *P* < 0.01), while Claudin-1 expression (*r* = −0.374, *P* < 0.01) negatively correlated with Eimeriidae. Jejunal IL-10 level positively correlated with Bacteroidaceae (*r* = 0.355, *P* < 0.01) and Lachnospiraceae (*r* = 0.388, *P* < 0.01), and negatively correlated with Eimeriidae (*r* = −0.323, *P* < 0.01). At genus level, jejunal IL-10 level positively correlated with *Bacteroides* (*r* = 0.355, *P* < 0.01), but negatively correlated with *Chlamydia* (*r* = −0.468, *P* < 0.01) and *Eimeria* (*r* = −0.323, *P* < 0.01). At species level, jejunal MDA level positively correlated with *E. tenella* (*r* = 0.322, *P* < 0.01). Jejunal SOD activity positively correlated with *Flavonifractor* sp. *An306* (*r* = 0.430, *P* < 0.01). Jejunal IL-10 level negatively correlated with *C. abortus* (*r* = −0.438, *P* < 0.01), *C. psittaci* (*r* = −0.407, *P* < 0.01) and *E. tenella* (*r* = −-0.332, *P* < 0.01), but positively correlated with *A. colihominis* (*r* = 0.427, *P* < 0.01). Additionally, jejunal Claudin-1 expression negatively correlated with *E. tenella* (*r* = −0.349, *P* < 0.01) (Figure 5A).

At day 42, at phylum level, jejunal T-AOC activity positively correlated with Tenericutes (*r* = 0.361, *P* < 0.01). Jejunal GPX activity positively correlated with Proteobacteria (*r* = 0.436, *P* < 0.01). At genus level, jejunal T-AOC activity negatively correlated with *Clostridium* (*r* = −0.361, *P* < 0.01). At species level, jejunal T-AOC activity positively correlated with *Clostridiales bacterium* (*r* = 0.391, *P* < 0.01) (Figure 5B).

### Functional Prediction of the Metagenomic Genes in the Cecal Contents

**FIGURE 6:**
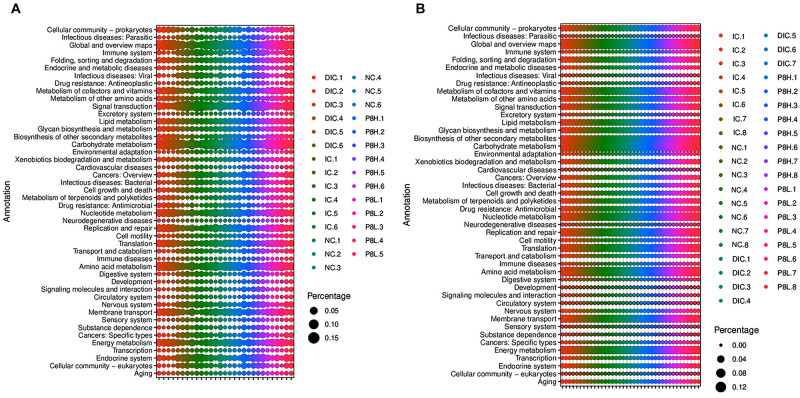
The Spearman correlation analyses of gut microbiota, coccidia with biochemical parameters of *Eimeria*-infected broilers. **(A)** Day 21, **(B)** day 42.

Predicted KEGG pathways from metagenomic sequences were profiled via PICRUSt. Differences in functional capacity were observed at day 21 but not day 42 (Supplementary Tables 3, 4 and Figure 6). Compared to the NC group, the metagenomes of the infected broilers showed enrichment of host genes modulating pathways involving neurodegenerative diseases, cardiovascular diseases, infectious diseases (viral) and infectious diseases (parasitic) (*P* < 0.05), but showed decrease of host genes modulating pathways involving energy metabolism, amino acid metabolism, translation, carbohydrate metabolism, glycan biosynthesis and metabolism, lipid metabolism and metabolism of cofactors and vitamins (*P* < 0.05). Moreover, compared to the IC group, the metagenomes in the P8L-treated broilers showed enrichment of host genes modulating pathways involving energy metabolism and replication repair (*P* < 0.05). Furthermore, the metagenomes in the DIC-treated broilers showed enrichment of host genes modulating pathways involving energy metabolism, amino acid metabolism, translation, replication and repair, carbohydrate metabolism, glycan biosynthesis and metabolism, lipid metabolism and metabolism of cofactors and vitamins (*P* < 0.05), but showed decrease of host genes modulating pathways involving neurodegenerative diseases, infectious diseases (viral) and infectious diseases (parasitic) (*P* < 0.05) in comparison to the IC group.

## Discussion

In the past decades, reports have proved that *Lactobacillus* were effective anti-coccidiosis candidates (Tierney et al., 2004; Dalloul et al., 2005; Kasornpikul et al., 2009). The anti-coccidiosis mechanisms of *Lactobacillus* may include the enhancement of cytokine levels, the elevation of anti-*Eimeria* antibody and the inhibition of oocyst shedding (Tierney et al., 2004; Travers et al., 2011). It was demonstrated that P8 provided protective immune response to broilers (Wang et al., 2015) and improved human gastrointestinal health. However, the anti-coccidiosis property and mechanisms of P8 are still unknown.

Adverse effects of coccidiosis include bloody diarrhea, intestinal lesions, depressed growth rate and, sometimes, high mortality. In this study, birds were experimentally infected with a mix of four *Eimeria* spp. and compared to non-infected and infected birds fed the P8 and DIC. The present study suggest that *Eimeria* infection caused obvious signs of coccidiosis, including the increased OPG, BDS, and CLS. While, supplementation of broilers with P8 and DIC resulted in lower OPG, BDS, and CLS values compared to the infected group. The above-mentioned results were in agreement with previous work (Giannenas et al., 2012) and implied that P8 may act as potential anticoccidial substances. Moreover, depressed growth performance was also found in *Eimeria-*infected broilers in this work during day 14 to day 42, as reflected by the lower ADG and higher F:G. However, groups supplemented with P8 and DIC had higher ADG and lower F:G than infected group. According to previous studies, broilers with coccidiosis also showed an impaired growth rate, while probiotics treatment improved the growth performance parameters (Lee et al., 2007; Ignatova et al., 2009; Giannenas et al., 2012; Sen et al., 2012). These data confirm that P8 supplementation protected broiler performance. Moreover, *Eimeria* infection caused an increased mortality during the whole period, but both doses of P8 and DIC down-regulated the mortality of boilers with coccidiosis. Similar results were found in the studies of Giannenas et al. (2012, 2014).

The reduction in growth performance due to the *Eimeria* challenge were caused by the damage to the intestinal mucosa (Ritzi et al., 2014). In our study, the mucosal architecture in terms of villus height and V/C was impaired by the *Eimeria* infection at day 21 and day 42, but was improved by P8H and DIC. The structure of the intestinal mucosa can reveal the intestinal health to some extent. Shorter jejunal villus height and lower ratio of villus height to crypt depth have been associated with stress (Song et al., 2014). Possible reason that explains the intestinal morphology change is that oocysts experience the excystation to generate the invasive sporozoites, which penetrate into epithelial cells of the intestinal mucosa, causing serious damage to the normal intestinal structure (Lai et al., 2018). Tight junctions are multi-protein complexes responsible for the regulation of permeability in the intestine via the modulation of its proteins (Sultana et al., 2013). The expression of tight junction proteins such as Occludin and Claudin-1 have been used to assess intestinal permeability in chickens (Chen et al., 2015; Li et al., 2015). *Eimeria* infection down-regulated mRNA expression of *occludin* (Schneiders et al., 2019) and *claudin-1* (Pham and Hatabu, 2021). Diamine oxidase is an intracellular enzyme catalyzing the oxidation of diamines and exists in high concentrations in the intestinal mucosa. Most diamine oxidase activity in the blood comes from the intestine. The serum diamine oxidase activity is reportedly proportional to the amount of intestinal diamine oxidase, therefore, it is a reliable marker of intestinal mucosal integrity (Kamiya et al., 2004). D-lactate is produced by some intestinal bacteria. Normally, serum levels of D-lactate are quite low. However, when the mucosa is injured and the intestinal permeability is increased, the elevated efflux of bacteria and their metabolisms, including D-lactate into the circulation occurred (Sun et al., 2001). In the present study, the increased intestinal permeability was also increased in the IC group, as reflected by decreased expression of Occludin and Claudin-1 in jejunal mucosa and increased diamine oxidase and D-lactate levels in serum. Probiotics and their effector molecules can influence the gut barrier and mucosal immunity by numerous methods including modulation of mucus production, reduction of bacterial adhesion, enhancement of tight junctions and cell survival, and induction of cytokines (O’Flaherty et al., 2010; Azad et al., 2018). These effects can be accomplished by influences on the intestinal barrier functions (Garcia-Lafuente et al., 2001). In the present study, high level P8 significantly improved the intestinal morphology at days 21 and 42, besides, both levels of P8 and DIC treatments up-regulated the expression of tight junction proteins and lowered the secretions of pro-inflammatory cytokines. The enhanced immune cell activity in *Eimeria-*infected chickens could cause the overproduction of free radicals (Allen, 1997), resulting in higher oxidative stress. Our data demonstrated that the decreased antioxidant capacity and increased oxidative stress were induced by the IC treatment, while, both levels of P8 and DIC down-regulated MDA levels in the condition of infection. Similar results were found in the study of Mengistu et al. (2021), in which the antioxidant capacity of chickens with coccidiosis was effectively elevated by probiotics. Besides, Dalloul et al. (2003) also found that *Lactobacillus*-based probiotics could regulate the local immune system of *Eimeria*-infected chickens.

Gut microbiota, the microbe population in the intestines, is one of the central defense components in the gastrointestinal tract against enteric pathogens, which works by modulating host responses to limit the colonization of pathogens (Rehman et al., 2007). Recent study also showed that the functional additives alleviated the impact of coccidiosis challenge on the microbiome of broilers (Vieira et al., 2020). By exploring gut microbial composition in chickens, we found that DIC could significantly increase the α-diversity in the infected broilers, as reflected by the Chao1 and Ace indices, however, P8L and P8H treatments had no significant effects on the α-diversity compared to the IC treatment. There is evidence suggesting that higher diversity microbiota is beneficial in chickens but the cause and effect relationships have not been elucidated (Stanley et al., 2014). However, other authors found that the overall microbial diversity is not significantly disturbed by feed additives (Neumann and Suen, 2015; Pourabedin et al., 2015). Furthermore, β-diversity indicated that the gut microbiota structure was altered by P8 and DIC treatments at day 21. By analyzing the taxa of gut microbiota, we noticed that the altered gut microbiota composition was more obvious at day 21 than that of day 42. At day 21, as for the major bacteria, broilers in the IC group had decreased Firmicutes and Clostridium abundances, and increased Chlamydiae, *Chlamydia*, *C. abortus*, and *C. psittaci* abundances. Although the P8 treatment had a tendency on reversing these changes, no significant statistical differences were found. However, it is worth noting that compared to the IC group, P8L and DIC increased the Firmicutes abundance and decreased the Chlamydiae abundance to the normal levels. Our results were in accordance with previous findings that *Eimeria* infection resulted in decreased Firmicutes (Huang et al., 2018), which may reduce the production of short-chain fatty acids (Duncan et al., 2007), leading to decreased intestinal barrier function (Stilling et al., 2016). Studies also suggest that probiotic supplementation could up-regulate Firmicutes (Del Campo et al., 2014; Stojanov et al., 2020). Unfortunately, no other studies have reported data on Chlamydiae in broilers with coccidiosis to serve for comparison with our results. It is also worth noting that at day 21, the Eimeriidae, *Eimeria* and *E. tenella* abundances were increased by *Eimeria* infection, however, low level P8 and DIC in the event of infection reversed these abundances to normal.

Positive correlation exists between gut microbial communities and bird performance (Torok et al., 2008), and gut dysbiosis in broiler chickens corresponded with gut inflammation and reduced growth and production (Teirlynck et al., 2011). Research indicated that coccidiosis was correlated with chicken gut pathology (Chang et al., 2016). With correlation analysis, we found that the SOD activity and IL-10 level positively correlated with the decreased abundance of Firmicutes in the IC group. Besides, the level of IL-10 and the expression of Claudin-1 negatively correlated with, but the level of MDA positively correlated with increased abundances of bacteria (Chalmydiae, *Chlamydia, C. abortus*, and *C. psittaci*) and coccidia (Eimeriidae, *Eimeria, E. tenella*) by *Eimeria* infection, indicating the challenge of *Eimeria* led to decreased antioxidant capacity, impaired tight junctions and lowered anti-inflammatory ability. On the contrary, the down-regulated Eimeriidae, *Eimeria, E. tenella* in P8L and DIC groups implied decreased oxidative stress and increased tight junctions as well as anti-inflammatory ability. At day 42, although correlation also existed between gut microbiota and biochemical parameters, the abundances of the major bacteria were not significantly altered by different treatments.

The change of gut microbiota may induce the altered microbial metabolic function (Zhu et al., 2021). Differences in functional capacity were observed at day 21 but not day 42. Predicted function of metagenomic genes in the cecal contents showed that the pathways involved in neurodegenerative diseases, cardiovascular diseases, infectious diseases (viral) and infectious diseases (parasitic) were enriched, while, pathways involved in energy metabolism, amino acid metabolism, translation, carbohydrate metabolism, glycan biosynthesis and metabolism, lipid metabolism and metabolism of cofactors and vitamins were decreased in cecal microbiota of broilers with coccidiosis at 21 days of age, implying that *Eimeria* infection may lead to diseases and had adverse effects on the nutrients metabolism. Similar to the DIC treatment, in the event of *Eimeria* infection, P8L treatment was predicted to induce a greater capacity for energy metabolism and replication repair. The greater capacity for energy metabolism is likely due to an increase in the level of Firmicutes as Firmicutes are related to energy metabolism and promote more efficient absorption of calories and subsequent weight gain (Koliada et al., 2017). Besides, Chlamydia infection causes host DNA damage and proliferation but impairs the DNA damage response (Chumduri et al., 2013), thus, the enhanced replication repair capacity of gut microbiota in P8L-treated broilers may be attributed to the decrease of Chlamydia abundance.

## Conclusion

The current study confirms that during the infection of *Eimeria*, P8 gave substantial reduction in oocysts shedding and improvement in growth performance and intestinal health in comparison to infected broilers, and these results approached to those of the DIC treatment. These beneficial effects may be partly due to the alteration of the gut microbiota as reflected by the correlation analysis between the gut microbiota, *Eimeria* and biochemical indices. The low level P8 had a more effective role in regulating the gut microbiota of broilers with coccidiosis than the high level P8. Functional predictions of metagenomic genes in the cecal contents suggest changes in pathways favoring diseases, and a reduction in nutrients metabolisms in *Eimeria*-infected broilers, and suggest a greater capacity for energy metabolism and replication repair in P8L-treated broilers. Furthermore, by comparing the data of anti-coccidial effect and gut microbiota at days 21 and 42, we found that the effects of P8 may be more effective in the early infection of coccidia. Thus, supplementation of 1 × 10^7^ cfu/g P8 in the early infection of coccidia provided the optimal effect.

## Data Availability Statement

The data presented in the study are deposited in the NCBI repository, accession number PRJNA683158.

## Ethics Statement

The animal study was reviewed and approved by Ethics and Animal Welfare Committee of Qingdao Agricultural University.

## Author Contributions

HL, KZ, and XH designed the study. XLv, KL, and XLi performed the research. YW analyzed data and wrote the manuscript. JZ and HL contributed to revision of the manuscript. All authors read and approved the final manuscript.

## Conflict of Interest

The authors declare that the research was conducted in the absence of any commercial or financial relationships that could be construed as a potential conflict of interest.

## Footnotes

1 http://qiime.org/

2 https://drive5.com/usearch/manua l/uclust_algo.html

3 https://www.r-project.org/

4 https://huttenhower.sph.harvard.edu/galaxy/
