# Supplementary material for: Protective Effect of Lactobacillus plantarum P8 on Growth Performance, Intestinal Health, and Microbiota in Eimeria-Infected Broilers
Source: Front Microbiol. 2021 Jul 9;12:705758. doi: 10.3389/fmicb.2021.705758 (PMC8299996; doi:10.3389/fmicb.2021.705758)
Supplement: Supplementary file 1 [file Table_1.DOCX]

Supplementary Material

**Table S1** Abundance of the metagenomic genes of broilers at day 21

|  | NC | IC | P8L | P8H | DIC | *P*-Value |
| --- | --- | --- | --- | --- | --- | --- |
| **Phylum** |  |  |  |  |  |  |
| Firmicutes | 38.826^a^ | 8.719^b^ | 19.041^ab^ | 9.245^b^ | 26.721^ab^ | 0.034 |
| Bacteroidetes | 14.141 | 8.846 | 14.020 | 9.306 | 18.358 | 0.565 |
| Chlamydiae | 1.660^c^ | 4.570^a^ | 3.034^abc^ | 4.216^ab^ | 1.807^bc^ | 0.041 |
| Proteobacteria | 1.461 | 1.223 | 1.872 | 1.368 | 1.714 | 0.041 |
| Actinobacteria | 1.004 | 1.069 | 0.971 | 0.774 | 0.767 | 0.787 |
| **Family** |  |  |  |  |  |  |
| Bacteroidaceae | 16.582 | 8.151 | 12.200 | 9.271 | 12.196 | 0.650 |
| Ruminococcaceae | 7.207 | 3.833 | 10.132 | 4.314 | 7.350 | 0.366 |
| Chlamydiaceae | 1.804 | 4.569 | 1.965 | 3.661 | 3.099 | 0.145 |
| Clostridiaceae | 3.784 | 0.755 | 4.942 | 1.919 | 2.076 | 0.080 |
| Lachnospiraceae | 3.595 | 1.256 | 3.499 | 1.579 | 2.538 | 0.303 |
| Eimeriidae | 0.115^b^ | 1.312^a^ | 0.013^b^ | 1.342^a^ | 0.152^b^ | 0.004 |
| **Genus** |  |  |  |  |  |  |
| *Bacteroides* | 12.269 | 8.129 | 12.686 | 8.693 | 16.525 | 0.609 |
| *Chlamydia* | 1.656^c^ | 4.569^a^ | 3.033^abc^ | 4.215^ab^ | 1.804^bc^ | 0.041 |
| *Clostridium* | 4.985^a^ | 0.213^c^ | 1.512^bc^ | 0.726^c^ | 3.334^ab^ | 0.002 |
| *Flavonifractor* | 2.247 | 1.689 | 2.544 | 0.618 | 1.278 | 0.463 |
| *Pseudoflavonifractor* | 1.172 | 0.874 | 0.851 | 0.837 | 1.105 | 0.976 |
| *Eimeria* | 0.010^b^ | 1.271^a^ | 0.017^b^ | 1.430^a^ | 0.111^b^ | 0.001 |
| **Species** |  |  |  |  |  |  |
| *Chlamydia abortus* | 1.196^b^ | 3.283^a^ | 2.219^ab^ | 2.901^ab^ | 1.226^b^ | 0.048 |
| *Bacteroides fragilis* | 1.026 | 0.818 | 1.037 | 0.905 | 0.858 | 0.988 |
| *Chlamydia psittaci* | 0.275^c^ | 0.763^a^ | 0.476^abc^ | 0.732^ab^ | 0.322^bc^ | 0.049 |
| *Anaerotruncus colihominis* | 0.592 | 0.110 | 0.899 | 0.047 | 0.826 | 0.222 |
| *Flavonifractor sp. An306* | 0.482 | 0.521 | 0.598 | 0.259 | 0.137 | 0.631 |
| *Eimeria tenella* | 0.005^b^ | 0.763^a^ | 0.006^b^ | 0.885^a^ | 0.068^b^ | 0.001 |

^a,b,c^ Mean value within a role with no common superscript differ significantly (P < 0.05). NC, control diet; IC, control diet + *Eimeria* infection; P8L, control diet containing 1 × 10^7^ cfu/g P8 + *Eimeria* infection; P8H, control diet containing 1 × 10^8^ cfu/g P8 + *Eimeria* infection; DIC, control diet + *Eimeria* infection + Diclazuril.

**Table S2** Abundance of the metagenomic genes of broilers at day 42

|  | NC | IC | P8L | P8H | DIC | *P-*Value |
| --- | --- | --- | --- | --- | --- | --- |
| **Phylum** |  |  |  |  |  |  |
| Firmicutes | 36.993 | 35.124 | 37.534 | 34.263 | 51.143 | 0.426 |
| Bacteroidetes | 25.320 | 24.894 | 23.330 | 23.796 | 18.098 | 0.658 |
| Proteobacteria | 1.572 | 1.473 | 1.455 | 1.509 | 1.555 | 0.903 |
| Actinobacteria | 0.734 | 0.815 | 0.798 | 0.779 | 0.884 | 0.268 |
| Tenericutes | 0.603 | 0.643 | 0.813 | 0.638 | 0.942 | 0.554 |
| **Family** |  |  |  |  |  |  |
| Rikenellaceae | 12.419 | 12.603 | 9.715 | 12.082 | 9.260 | 0.792 |
| Ruminococcaceae | 7.074 | 8.032 | 8.311 | 6.947 | 10.194 | 0.040 |
| Bacteroidaceae | 7.802 | 6.950 | 8.823 | 5.781 | 3.106 | 0.285 |
| Clostridiaceae | 6.234 | 6.086 | 6.084 | 5.682 | 8.242 | 0.197 |
| Lachnospiraceae | 6.425 | 5.201 | 5.389 | 4.908 | 5.516 | 0.571 |
| **Genus** |  |  |  |  |  |  |
| *Alistipes* | 12.358 | 12.548 | 9.658 | 12.031 | 9.230 | 0.793 |
| *Bacteroides* | 7.392 | 6.249 | 8.244 | 5.512 | 3.012 | 0.268 |
| *Clostridium* | 4.979 | 5.014 | 5.374 | 4.607 | 7.454 | 0.121 |
| *Lachnoclostridium* | 2.706 | 1.921 | 1.894 | 1.637 | 1.983 | 0.323 |
| *Subdoligranulum* | 0.849 | 1.464 | 1.443 | 1.045 | 2.981 | 0.056 |
| **Species** |  |  |  |  |  |  |
| *Chlamydia abortus* | 1.778 | 0.770 | 2.514 | 1.059 | 1.930 | 0.355 |
| *Bacteroides fragilis* | 0.607 | 1.144 | 0.163 | 0.763 | 2.492 | 0.064 |
| *Chlamydia psittaci* | 0.891 | 0.676 | 0.655 | 0.572 | 0.604 | 0.760 |
| *Anaerotruncus colihominis* | 0.599 | 0.654 | 0.539 | 0.406 | 0.709 | 0.667 |
| *Flavonifractor sp. An306* | 0.770 | 0.581 | 0.209 | 0.654 | 0.241 | 0.499 |

NC, control diet; IC, control diet + *Eimeria* infection; P8L, control diet containing 1 × 10^7^ cfu/g P8 + *Eimeria* infection; P8H, control diet containing 1 × 10^8^ cfu/g P8 + *Eimeria* infection; DIC, control diet + *Eimeria* infection + Diclazuril.

**Table S3** Functional prediction of the metagenomic genes of broilers at day 21

|  | NC | IC | P8L | P8H | DIC | SEM | *P*-value |
| --- | --- | --- | --- | --- | --- | --- | --- |
| Aging | 1271.33±226.38 | 959.17±202.77 | 1118.6±246.96 | 1053±322.62 | 1269.33±262.94 | 49.86284 | 0.181 |
| Cellular community - eukaryotes | 1326.17±895^b^ | 2729.83±136.93^a^ | 1864.8±1259.77^ab^ | 2692.83±270.05^a^ | 1572.83±1191.03^b^ | 185.15299 | 0.026 |
| Endocrine system | 5254.17±1407.31 | 7146.67±447.33 | 6017.2±2509.77 | 7314±694.12 | 5750.17±2083.86 | 312.83263 | 0.14 |
| Transcription | 931.5±141.04 | 835.83±149.1 | 845.4±199.27 | 924.83±242.96 | 953.5±134.05 | 31.79497 | 0.716 |
| Energy metabolism | 13511.67±4929.69^b^ | 5097.5±3630.66^a^ | 10444.8±4497.08^ab^ | 6133.5±5254.8^a^ | 12847.67±5762.5^b^ | 1067.19409 | 0.018 |
| Cancers: Specific types | 1527.17±854.15^b^ | 2781±73.39^a^ | 2011.4±1194.5^ab^ | 2780.17±181.05^a^ | 1738.67±1133.06^ab^ | 172.49275 | 0.035 |
| Substance dependence | 716.33±487.02^b^ | 1527.67±83.31^a^ | 1017.4±705.09^ab^ | 1505.5±170.41^a^ | 851.67±669.23^b^ | 104.94785 | 0.020 |
| Sensory system | 501.67±319.35^b^ | 1020.17±42.06^a^ | 682.8±454.46^ab^ | 1009.83±96.6^a^ | 585±445.91^b^ | 68.23019 | 0.022 |
| Membrane transport | 12829.83±4833.31^b^ | 4848.33±3775.77^a^ | 9728.4±4326.63^ab^ | 5838.33±5155.27^a^ | 12312.5±5474.69^b^ | 1032.33653 | 0.021 |
| Nervous system | 2471.17±1023.91^b^ | 4072.83±178.98^a^ | 3072.2±1594.37^ab^ | 4070.67±259.71^a^ | 2794.33±1441.78^ab^ | 220.17494 | 0.039 |
| Circulatory system | 507.5±339.8^b^ | 1068.67±51.83^a^ | 710±477^ab^ | 1066.17±86.52^a^ | 617.17±467.61^b^ | 72.29415 | 0.018 |
| Signaling molecules and interaction | 1015.5±570.5^b^ | 1832.17±56.01^a^ | 1323.4±796.61^ab^ | 1813.5±128.63^a^ | 1143.5±742.66^ab^ | 113.46961 | 0.042 |
| Development | 545.17±364.88^b^ | 1090.5±45.87^a^ | 752.4±504.87^ab^ | 1081.67±88.15^a^ | 636.5±477.62^ab^ | 73.63519 | 0.031 |
| Digestive system | 1044±619.56^b^ | 2030.67±99.52^a^ | 1421.4±897.18^ab^ | 2020.33±175.54^a^ | 1242.5±843.57^ab^ | 130.36162 | 0.027 |
| Amino acid metabolism | 21863.5±7614.88^a^ | 8937.5±6202.59^b^ | 17341.8±7136.21^ab^ | 10538±8589.16^b^ | 21049.17±8833^a^ | 1679.78218 | 0.022 |
| Immune diseases | 473.67±176.99 | 712±24.74 | 551±255.72 | 707.67±42.83 | 504.5±235.48 | 35.31499 | 0.063 |
| Transport and catabolism | 1523.5±349.8 | 1995.83±139.11 | 1706.4±586.63 | 2035.33±193.97 | 1650.83±537.93 | 77.77676 | 0.139 |
| Translation | 13142.67±4844.36^a^ | 4629.67±2697.41^b^ | 9461.4±4041.47^ab^ | 6020.33±4738^b^ | 12022.33±5500.59^a^ | 998.29926 | 0.012 |
| Cell motility | 1823±353.9 | 1375±407.37 | 1708.8±339.44 | 1532.5±504.24 | 1612.17±277.65 | 72.44618 | 0.355 |
| Replication and repair | 14371±5285.77^a^ | 4731.33±3288.27^b^ | 10663±4456.42^ab^ | 6172.5±5357.86^b^ | 13183.17±6162.98^a^ | 1130.36164 | 0.01 |
| Neurodegenerative diseases | 150±51.14^c^ | 230.67±8.76^ab^ | 177±78.49^bc^ | 240.67±14.57^ab^ | 173.17±63.8^c^ | 10.88966 | 0.017 |
| Nucleotide metabolism | 15380.17±5452.38^a^ | 5656.33±3765.3^b^ | 11688.8±4744.08^ab^ | 7046±5765.42^b^ | 14356.33±6344.51^a^ | 1177.59274 | 0.013 |
| Drug resistance: Antimicrobial | 3999.5±1536.4^a^ | 1282.5±1045.97^b^ | 3014±1361.79^ab^ | 1618.5±1588.29^b^ | 3704.67±1806.34^a^ | 331.75161 | 0.013 |
| Metabolism of terpenoids and polyketides | 3351.17±1261.84^a^ | 1165.5±875^b^ | 2511.8±1142.24^ab^ | 1455.17±1332.08^b^ | 3100.83±1477.4^a^ | 270.69062 | 0.017 |
| Cell growth and death | 3343.33±560.12 | 2690.83±485.2 | 3049±686.31 | 2945.17±811.18 | 3290.83±630.17 | 119.54204 | 0.413 |
| Infectious diseases: Bacterial | 2591.17±393.41 | 2738.5±337.64 | 2624.8±734.72 | 2858.67±464.49 | 2661.67±569.67 | 89.28004 | 0.901 |
| Cancers: Overview | 4055.83±1007.22 | 5131.5±377.21 | 4446.6±1650.61 | 5235.83±523.86 | 4295.17±1377.64 | 204.83984 | 0.257 |
| Cardiovascular diseases | 452.5±296.43^b^ | 924.5±50.46^a^ | 635.8±434.05^ab^ | 915.33±86.09^a^ | 544.33±401.51^ab^ | 62.40813 | 0.029 |
| Xenobiotics biodegradation and metabolism | 2990.33±1014.91^a^ | 1244.33±772.59^b^ | 2348±910.97^ab^ | 1509.33±1152.79^b^ | 2846.83±1177.23^a^ | 221.19422 | 0.021 |
| Environmental adaptation | 782.5±241.6^b^ | 1109.67±75.43^a^ | 924.8±377.12^ab^ | 1128.5±103.39^a^ | 865.83±327.47^ab^ | 50.02006 | 0.094 |
| Carbohydrate metabolism | 35976±12812.31^a^ | 13967.17±9641.85^b^ | 28122.6±11467.7^ab^ | 16859.5±14144.11^b^ | 34114.67±14665.06^a^ | 2778.75837 | 0.018 |
| Biosynthesis of other secondary metabolites | 4117.83±1442.85^a^ | 1572.33±1068.85^b^ | 3195.8±1330.06^ab^ | 1866.17±1569.21^b^ | 3870±1661.16^a^ | 316.36572 | 0.014 |
| Glycan biosynthesis and metabolism | 5345.5±1736.01^a^ | 2241.5±1192.68^b^ | 4251.8±1396.44^ab^ | 2671.67±1853.36^b^ | 4952.67±1982.78^a^ | 371.66012 | 0.012 |
| Lipid metabolism | 7919.5±2179.83^a^ | 4230.5±1711.29^c^ | 6616.8±1914.55^abc^ | 4909.5±2735.49^bc^ | 7602.83±2442.88^ab^ | 475.95301 | 0.032 |
| Excretory system | 331.17±164.62^a^ | 604.33±20.1^b^ | 426.4±222.05^ab^ | 603.67±34.56^b^ | 390.33±219.43^a^ | 34.3833 | 0.014 |
| Signal transduction | 12915.83±2551.95 | 15216.67±1568.39 | 13791.6±4480.83 | 15659.67±2057.97 | 13688.67±3514.57 | 542.80871 | 0.481 |
| Metabolism of other amino acids | 4568.83±1546.7^a^ | 1930.5±1201.79^b^ | 3602.4±1386.42^ab^ | 2252.83±1691.73^b^ | 4368.33±1766.26^a^ | 335.7594 | 0.018 |
| Metabolism of cofactors and vitamins | 11116±3975.03^a^ | 4355.33±3125.05^b^ | 8674.8±3828.77^ab^ | 5202.83±4385.09^b^ | 10686.33±4708.49^a^ | 876.58189 | 0.021 |
| Drug resistance: Antineoplastic | 650.33±342.31 | 1221.5±56.34^a^ | 872.6±554.3^a^ | 1225.17±115.04^a^ | 760.17±487.74^a^ | 76.62695 | 0.028 |
| Infectious diseases: Viral | 1682.5±1155.34^b^ | 3577.17±194.71^a^ | 2348±1624.67^ab^ | 3519.67±390.58^a^ | 1977±1571.62^b^ | 245.67439 | 0.019 |
| Endocrine and metabolic diseases | 1837.5±396.22 | 2251.17±190.01 | 2009.6±754.85 | 2304.17±267.6 | 1955.17±570.18 | 87.20814 | 0.385 |
| Folding, sorting and degradation | 6017.83±1691.07^a^ | 3148.33±1306.24^c^ | 4894.4±1418.1^abc^ | 3642.5±1989.88^bc^ | 5735.33±1903.8^ab^ | 362.97203 | 0.026 |
| Immune system | 2277.33±1254.58^b^ | 4181.5±162.66^a^ | 3035.8±1803.46^ab^ | 4171.33±326.13^a^ | 2618.83±1685.54^ab^ | 258.68737 | 0.034 |
| Global and overview maps | 28663.33±10531.85^a^ | 10413.5±7836.68^b^ | 22083.4±9789.56^ab^ | 12808.83±11514.1^b^ | 27177.5±12366.72^a^ | 2305.10566 | 0.018 |
| Infectious diseases: Parasitic | 911.17±582.25^b^ | 1864.17±85.62^a^ | 1263±849.84^ab^ | 1835.5±177.3^a^ | 1090±781.19^b^ | 123.39495 | 0.022 |
| Cellular community - prokaryotes | 5628.5±2218.88^a^ | 1825.83±1549.55^b^ | 4185.6±1964.14^ab^ | 2306.5±2294.56^b^ | 5422.17±2633.46^a^ | 478.99159 | 0.014 |

^a,b,c^ Mean value within a role with no common superscript differ significantly (P < 0.05). NC, control diet; IC, control diet + *Eimeria* infection; P8L, control diet containing 1 × 10^7^ cfu/g P8 + *Eimeria* infection; P8H, control diet containing 1 × 10^8^ cfu/g P8 + *Eimeria* infection; DIC, control diet + *Eimeria* infection + Diclazuril.

**Table S4** Functional prediction of the metagenomic genes of broilers at day 42

|  | NC | IC | P8L | P8H | DIC | SEM | *P*-value |
| --- | --- | --- | --- | --- | --- | --- | --- |
| Aging | 2961.75±531.59 | 3223.63±110.01 | 3095.25±410.5 | 2797.38±588.91 | 3157.14±286.22 | 69.07 | 0.307 |
| Cellular community - eukaryotes | 40±46.6 | 54±61.51 | 24.5±17.16 | 47.88±51.48 | 28.14±32.38 | 7.04 | 0.656 |
| Endocrine system | 6229.38±1140.16 | 6894.25±227.83 | 6536±973.22 | 5888.63±1247.13 | 6774.29±643.54 | 153.12 | 0.217 |
| Transcription | 2038.38±430.16 | 2243.38±64.05 | 2117.63±345.79 | 1927.25±464.43 | 2231.14±205.56 | 54.97 | 0.329 |
| Energy metabolism | 39692.88±6382.62 | 43760.38±1269.02 | 41399±5044.06 | 37281.25±7647.79 | 41602.14±4310.53 | 892.90 | 0.2 |
| Cancers: Specific types | 598.5±76.4 | 666.5±58.59 | 606.5±80.27 | 588±96.25 | 597.86±67.11 | 12.55 | 0.281 |
| Substance dependence | 21.75±23.89 | 29.25±30.65 | 12±10.32 | 21.88±27.51 | 15.29±14.64 | 3.61 | 0.62 |
| Sensory system | 16.5±13.08 | 21.38±21.19 | 13.88±6.6 | 18.25±16.18 | 14.71±11.54 | 2.26 | 0.856 |
| Membrane transport | 35412.88±5674.99 | 39003±791.83 | 36400.75±4206.37 | 32779.25±6917.05 | 36632.14±3808.25 | 795.38 | 0.157 |
| Nervous system | 2089.13±232.41 | 2280.63±94.65 | 2141.88±175.89 | 1989.63±292.63 | 2109.57±229.65 | 36.06 | 0.131 |
| Circulatory system | 28.88±19.41 | 32.75±24.25 | 23.25±9.33 | 31.75±19.42 | 21.43±12.84 | 2.82 | 0.663 |
| Signaling molecules and interaction | 243.63±63.61 | 288.63±44.09 | 243.25±58.43 | 227.38±75.91 | 275±51.35 | 9.79 | 0.255 |
| Development | 15.5±21.37 | 22.75±29.03 | 10±10.03 | 18.13±23.11 | 10.14±15.87 | 3.28 | 0.723 |
| Digestive system | 289.5±61.2 | 335.63±37.22 | 306.5±59.97 | 283.38±62.42 | 337.43±36.25 | 8.84 | 0.165 |
| Amino acid metabolism | 62901.88±8682.75 | 68678.13±1847.5 | 65072.63±7009.54 | 58871.13±11139.5 | 64352.43±7077.3 | 1290.97 | 0.18 |
| Immune diseases | 459.38±83.43 | 493.38±35.94 | 470.5±71.72 | 430.88±93.04 | 479.57±62.26 | 11.48 | 0.506 |
| Transport and catabolism | 2268.25±340.68 | 2441.38±106.27 | 2367.63±314 | 2165.25±347.49 | 2370±228.77 | 45.79 | 0.356 |
| Translation | 37508.88±8133.51 | 41702.38±1295.53 | 39466.88±6218.35 | 35198.5±9099.23 | 40902.71±4630 | 1062.25 | 0.298 |
| Cell motility | 2141±385.79 | 2461.13±160.94 | 2426.88±367.19 | 2212.88±382.55 | 2181.86±284.22 | 54.28 | 0.197 |
| Replication and repair | 40385.63±8414.17 | 44841.63±1352.25 | 42491.75±6806.89 | 38060.13±9240.47 | 44253.29±4384.05 | 1099.86 | 0.269 |
| Neurodegenerative diseases | 163.38±23.27 | 167.63±9.09 | 168.5±20.92 | 152.13±31.3 | 172±20.24 | 3.54 | 0.457 |
| Nucleotide metabolism | 43866.25±8085.85 | 48424.63±1435.95 | 45904.88±6456.15 | 41230±9235.37 | 47026.14±4886.41 | 1088.19 | 0.249 |
| Drug resistance: Antimicrobial | 11690±1997.59 | 12887.75±339.97 | 12203.13±1654.37 | 10962.88±2407.23 | 12385.86±1214.55 | 277.78 | 0.227 |
| Metabolism of terpenoids and polyketides | 9634.75±1670.85 | 10585.88±343.32 | 10103.63±1388.52 | 9012.13±1950.47 | 10171.43±1054.61 | 230.36 | 0.238 |
| Cell growth and death | 7018.75±1465.85 | 7800.75±252.36 | 7371.75±1193.57 | 6621.5±1600.95 | 7707.29±767 | 191.68 | 0.267 |
| Infectious diseases: Bacterial | 4428.88±771.7 | 4887.5±166.49 | 4652.38±600.05 | 4229.5±868.02 | 4690.71±418.24 | 101.46 | 0.283 |
| Cancers: Overview | 5324.38±858.14 | 5834.88±176.98 | 5479.63±768.75 | 4993.88±1049.29 | 5601.14±576.6 | 122.81 | 0.258 |
| Cardiovascular diseases | 27.88±20.07 | 30.63±21.76 | 19.75±8.63 | 29.5±17.49 | 20.14±11.02 | 2.65 | 0.561 |
| Xenobiotics biodegradation and metabolism | 8096.38±1303.24 | 8938.38±225.64 | 8389.13±1076.62 | 7515.75±1587.86 | 8356±979.02 | 187.83 | 0.183 |
| Environmental adaptation | 1023.25±140.94 | 1127.75±32.94 | 1057.25±117.94 | 985.13±144.82 | 1035.57±91.16 | 18.91 | 0.169 |
| Carbohydrate metabolism | 101078±16203.17 | 111612.25±2953.4 | 105367±12436.13 | 95312±19013.18 | 106295.29±10947.35 | 2237.27 | 0.192 |
| Biosynthesis of other secondary metabolites | 11719.38±1752.1 | 12933.75±445.71 | 12265.38±1479.24 | 10965.5±2135.29 | 12205.29±1203.55 | 254.28 | 0.145 |
| Glycan biosynthesis and metabolism | 15238.38±2748.91 | 16797.75±557.32 | 16136.25±2345.09 | 14438±3097.47 | 16345.86±1515.9 | 369.93 | 0.262 |
| Lipid metabolism | 20112.13±3558.19 | 22130.88±615.31 | 20920.25±2908.72 | 18894.88±4080.2 | 21398±2202.14 | 482.57 | 0.253 |
| Excretory system | 249.63±29.6 | 278.88±18.33 | 261.88±21.44 | 241±29.6 | 256.86±32.09 | 4.54 | 0.079 |
| Signal transduction | 18404.88±2917.72 | 20288.25±552.76 | 19020.75±2417.76 | 17222.38±3623.89 | 19263.14±1872.05 | 415.90 | 0.195 |
| Metabolism of other amino acids | 12653.88±1943.06 | 13914.63±347.16 | 13144.75±1602.13 | 11849.25±2318.84 | 13168.14±1381.91 | 275.90 | 0.181 |
| Metabolism of cofactors and vitamins | 32656.5±4680.99 | 35720.75±1124.34 | 33899.38±3993.91 | 30645.75±5792.4 | 33424.29±3859.05 | 690.98 | 0.208 |
| Drug resistance: Antineoplastic | 292.5±69.27 | 322.25±30.12 | 302.88±65.44 | 274.5±76.82 | 338.14±31.15 | 9.57 | 0.267 |
| Infectious diseases: Viral | 105.75±66.33 | 114.75±88.87 | 91.38±43.95 | 116.75±75.52 | 86.86±60.72 | 10.62 | 0.88 |
| Endocrine and metabolic diseases | 2565.25±460.93 | 2854.13±102.15 | 2709.38±367.7 | 2431.25±519.21 | 2782.71±265.51 | 62.19 | 0.193 |
| Folding, sorting and degradation | 15710.63±2908.03 | 17290.63±458.04 | 16428.75±2325.19 | 14774±3306.79 | 16796.57±1626.1 | 386.62 | 0.267 |
| Immune system | 989.13±126.82 | 1100.13±105.83 | 994.63±145.37 | 960.38±160.68 | 1007.86±108.92 | 21.43 | 0.291 |
| Global and overview maps | 82343.63±12809.45 | 90462.25±2417.49 | 85539.5±10245.53 | 77287±15946.15 | 85881.71±9301.86 | 1833.30 | 0.217 |
| Infectious diseases: Parasitic | 244.13±40.51 | 267.25±49.33 | 249.75±37.2 | 244.75±49.85 | 243.71±49.52 | 7.04 | 0.82 |
| Cellular community - prokaryotes | 16384.25±2846.52 | 17929.13±438.63 | 16869.63±2280.96 | 15107.13±3458.47 | 17023.43±1928.57 | 397.86 | 0.241 |

NC, control diet; IC, control diet + *Eimeria* infection; P8L, control diet containing 1 × 10^7^ cfu/g P8 + *Eimeria* infection; P8H, control diet containing 1 × 10^8^ cfu/g P8 + *Eimeria* infection; DIC, control diet + *Eimeria* infection + Diclazuril.
